# Comparative Evaluation of Conventional Methods and Matrix-Assisted Laser Desorption/Ionization Time-of-Flight Mass Spectrometry (MALDI-TOF MS) for Uropathogen Identification in Catheter-Associated Urinary Tract Infections in ICU Patients

**DOI:** 10.7759/cureus.83134

**Published:** 2025-04-28

**Authors:** Anuj Kumar, Riddhi Singh, Ravindra Gupta, Nikhil Raj, RajKumar Kalyan

**Affiliations:** 1 Microbiology, King George's Medical University, Lucknow, IND; 2 Microbiology, Dr. Ram Manohar Lohia Institute of Medical Sciences, Lucknow, IND; 3 Department of Orthopaedic Surgery, Hind Institute of Medical Sciences, Sitapur, IND

**Keywords:** antibiotic resistance, candida, cauti, enterococcus, icu infections, maldi-tof ms

## Abstract

Background

Catheter-associated urinary tract infections (CAUTIs) are a significant concern in ICU patients, often leading to complications such as bloodstream infections, urosepsis, prolonged hospitalization, and increased mortality, particularly due to delayed diagnosis and rising antibiotic resistance. For therapy to be effective, the causing pathogens must be identified quickly and accurately. This study compares conventional microbiological methods with Matrix-Assisted Laser Desorption/Ionization Time-of-Flight Mass Spectrometry (MALDI-TOF MS) for identifying urinary isolates in CAUTI cases and examines their antibiotic susceptibility patterns.

Methods

Over one year, we analyzed 780 catheterized urine samples from ICU patients at a tertiary care hospital. All samples were processed using standard culture techniques and MALDI-TOF MS for pathogen identification. Antibiotic susceptibility testing was performed according to Clinical and Laboratory Standards Institute (CLSI) guidelines. Descriptive statistics were used for data summarization, and Chi-square test assessed associations between risk factors and CAUTI occurrence, with agreement between MALDI-TOF MS and conventional identification methods evaluated by Cohen’s Kappa analysis. The Chi-square test's statistical significance was set at *p* < 0.05.

Results

Out of 780 samples, 156 (20%) showed significant bacterial or fungal growth. The most common pathogens were *Candida* species (56.4%), *Enterococcus* (17.9%), and *Escherichia coli* (12.2%). MALDI-TOF MS demonstrated good accuracy, with misclassifications in 12 isolates (7.7%) mainly involving misidentification in *Enterococcus* and *Candida.* Despite these discrepancies, a strong agreement was observed between the two methods, with a Cohen’s Kappa value of 0.787. High resistance was observed against fluoroquinolones and cephalosporins, while fosfomycin and linezolid remained effective against *Enterococcus* spp.

Conclusion

MALDI-TOF MS enhances the speed and accuracy of pathogen identification, making it a valuable tool for managing CAUTI cases. The increasing antibiotic resistance observed in this study highlights the urgent need for targeted treatment strategies and stricter infection control measures in ICU settings.

## Introduction

Nearly half of all hospital-acquired infections are catheter-associated urinary tract infections (CAUTIs), making them the most prevalent hospital-acquired infection in the world [1]. One of the main causes of the high incidence of CAUTI in healthcare facilities is the extensive use of indwelling catheters [2]. The Centers for Disease Control and Prevention (CDC) defines a CAUTI as a UTI that occurs in a patient who has had a urinary catheter inserted and left in place for at least 48 hours [3]. A study conducted in 40 Indian hospitals from 2004 to 2013 revealed an average prevalence of 2.1 CAUTI cases per 1000 catheter days [4].

Identification of the causative microorganisms is crucial for effective treatment and monitoring of nosocomial as well as community infections. Clinical laboratories traditionally rely on conventional phenotypic methods such as Gram staining, growth on nutrient media, colony morphology, and various biochemical tests to classify clinical isolates to the genus level. For the quick and accurate detection of pathogens in UTIs, molecular approaches such as 16S RNA sequencing, biosensors, and several polymerase chain reaction (PCR)-based technologies have recently become more and more popular [5]. Despite their excellent performance characteristics, these methods are labour-intensive, require skilled technical staff, and are often not feasible due to their complexity and high cost. Routine biochemical and phenotypic methods, including culture and Gram staining, remain essential in clinical microbiology. Even though it usually takes more than two days to identify bacteria and administer the right medication, urine sample culture is still the gold standard for diagnosing UTIs.

Matrix-Assisted Laser Desorption/Ionization Time-of-Flight Mass Spectrometry (MALDI-TOF MS) is increasingly recognized as a transformative tool in diagnostic microbiology due to its speed and precision [6]. This innovative technique allows for the rapid and accurate identification of microorganisms, making it a valuable tool for diagnosing infections in critically ill patients [7]. Compared to traditional methods such as biochemical tests and culture-based identification, MALDI-TOF MS is faster, more efficient, and more cost-effective. 

The primary objective of this study was to compare the accuracy of conventional microbiological methods with MALDI-TOF MS for identifying uropathogens in CAUTI cases among Intensive Care Unit (ICU) patients. The secondary objectives included determining the time-to-identification for both methods, analyzing the antibiotic susceptibility patterns of the isolated pathogens, and assessing the concordance between conventional and MALDI-TOF MS methods at both the genus and species levels.

We hypothesized that MALDI-TOF MS would provide more accurate species-level identification compared to conventional methods, especially for difficult-to-identify organisms such as non-albicans Candida species. By evaluating both identification accuracy and antibiotic susceptibility patterns, the study aims to highlight the clinical utility and potential advantages of MALDI-TOF MS in the rapid and precise diagnosis of CAUTI pathogens in critically ill patients.

## Materials and methods

This retrospective observational study was conducted at the Postgraduate Department of Microbiology, King George’s Medical University, over a period of one year (September 2020-2021). A total of 780 catheterised urine samples from patients admitted to ICUs were analysed. The Institutional Ethics Committee of King George’s Medical University issued approval 921/Ethics/2020.

Inclusion criteria

Patients admitted to ICUs with an indwelling urinary catheter in place for more than 48 hours, aged 18 years and above, and exhibiting symptoms of UTI such as fever, chills, dysuria, suprapubic pain, or cloudy/malodorous urine were included. Urine samples were obtained via aseptic aspiration from the catheter tubing and transported immediately to the microbiology laboratory.

Exclusion criteria

Patients with a documented history of UTI within the last two weeks before admission or those receiving antibiotic therapy at the time of enrollment were excluded. This ensured that only newly acquired infections were analyzed, minimizing potential biases in the study results.

Urine samples from catheterized patients were collected in sterile containers by aspirating urine directly from the section of the catheter tubing closest to the clamp, after cleaning the area with alcohol and puncturing the tubing with a sterile syringe and needle. The samples were immediately transported to the Microbiology laboratory. The samples were processed using standard laboratory techniques, which included microscopy, culture identification, and antibiotic susceptibility testing. Macroscopic examination of urine samples was performed to assess turbidity, color, and other gross characteristics. Urine microscopy was performed on catheter urine specimen. Culture was set up on cysteine-, lactose-, and electrolyte-deficient (CLED) agar. For 18 to 24 hours, all of the culture plates were incubated aerobically at 37°C. The colony morphology, Gram stain, and biochemical characterization of all the significant culture-positive isolates were used for both conventional and MALDI-TOF MS final identification. Using CLSI guideline 2020 M100, the Kirby-Bauer disc-diffusion technique was used to test for antimicrobial susceptibility on Muller-Hinton agar.

Statistical analysis

Descriptive statistics were used to summarise the number of positive cultures, age and gender distribution, mean and standard deviation of age, catheterisation duration, clinical symptoms, pathogen distribution, and antibiotic sensitivity patterns. Chi-square test was applied to analyse the association between risk factors such as gender, age groups, and diabetes mellitus with the occurrence of CAUTI. Agreement between conventional identification and MALDI-TOF MS was assessed using Cohen’s Kappa analysis. P values less than 0.05 were regarded as statistically significant.

## Results

**Figure 1:**
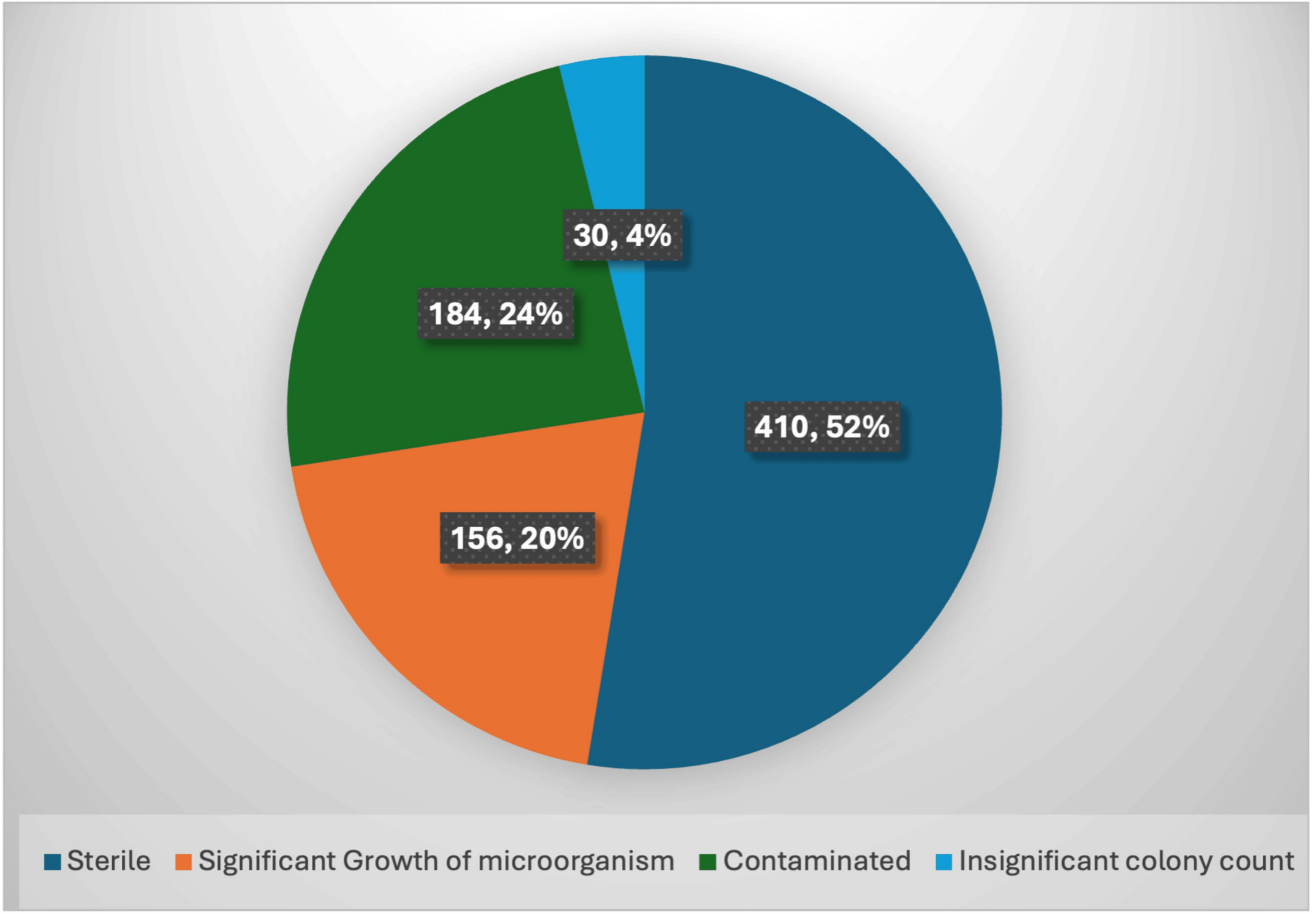
Proportion of Catheterized Urine Samples Showing Significant Growth

Out of the total of 780 catheterised urine samples received from different ICUs, only 156 showed significant growth of bacteria (Figure 1). On analysing the age and gender distribution (Table 1), among patients aged 18-40 years, females constituted the majority. Further, between 41-65 years, the majority of the population was male while in the above 65 years of age group, the majority of the population was also male. The age range of study participants was between 18-87 years with a mean age of 48.11±18.94. Maximum patients showed growth in urine culture between two to seven days’ time interval of catheterization (53.20%), followed by eight to 14 days of time interval (28.20%) (Table 2).

**Table 1:** Age- and Gender-Wise Distribution of the Study Population

Age	MALE	FEMALE	RANGE	MEAN ± SD
	N (%)	N (%)		
18-40 years (n=63)	30 (32.3)	33 (52.4)	18-87 years	48.11±18.94
41-65 years (n=52)	34 (36.6)	18 (28.6)		
>65 years (n=41)	29 (31.2)	12 (19.1)		
TOTAL	93	63		

**Table 2:** Correlation Between Time Interval Between Catheterization and Appearance of Growth in Urine Culture

Time Interval (days)	Number of samples (%)
2-7	83 (53.2)
8-14	44 (28.2)
15-21	14 (9.0)
22-28	15 (9.6)
TOTAL	156

**Figure 2:**
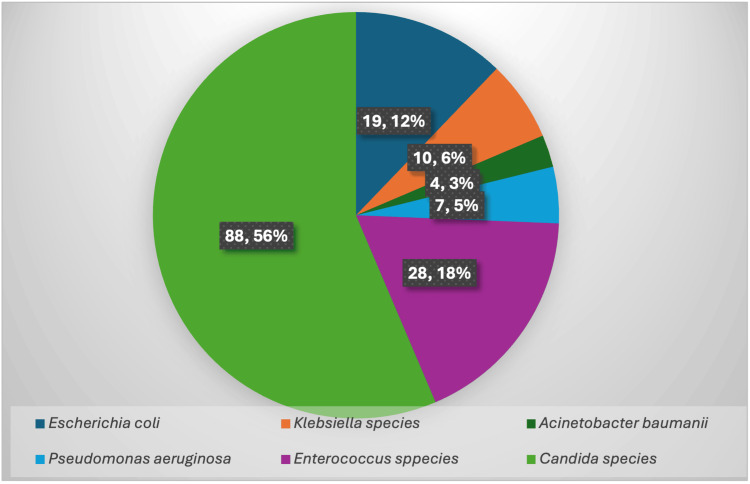
Distribution of Pathogens Isolated From CAUTI Cases CAUTI: Catheter-Associated Urinary Tract Infection

The most common presenting symptom in patients with CAUTI was fever, seen in 156 (100%) patients, followed by chills seen in 14 (8.97%) and pain in the lower abdomen seen in 13 (8.33%) patients. While analysing the risk factors of CAUTI patients, the prominent risk factors observed were female gender, age above 65 years and presence of diabetes mellitus (Table 3). The distribution of pathogens isolated from samples is shown in Figure 2.

**Table 3:** Risk Factors in CAUTI Patients CAUTI: Catheter-Associated Urinary Tract Infection

Risk Factors		Total number of patients enrolled	Number of patients with significant growth	p-value
Gender	Female	253	63 (24.90%)	Χ^2^=5.622, P=0.0177
	Male	527	93 (17.65%)	
Age >65 years		162	41 (25.31%)	Χ^2^=39.5, P<0.0001
History of diabetes mellitus		180	28 (15.56%)	χ^2^=64.1, P<0.0001

On comparative analyses of the results we found 12 discrepancies. The majority of discrepancies amongst the isolates by the conventional method versus MALDI-TOF MS were found in Enterococcus faecalis identified as Enterococcus faecium by MALDI-TOF, followed by Candida glabrata identified as Candida auris by MALDI-TOF and Candida tropicalis identified as Candida rugosa by MALDI-TOF, respectively (Table 4). On quantifying the agreement between identification by the conventional method versus MALDI-TOF with Cohen’s Kappa analysis, we found a strong agreement (Kappa=0.787).

**Table 4:** Discrepancies Amongst the Isolates by Conventional Method Versus MALDI-TOF MS MALDI-TOF MS: Matrix-Assisted Laser Desorption/Ionization Time-of-Flight Mass Spectrometry In all cases listed, conventional methods provided the correct identification, while MALDI-TOF misidentified the organisms, likely due to database limitations or spectral overlap.

DISCREPANCY	NUMBER	PERCENTAGE
Klebsiella pneumonia identified as Klebsiella oxytoca by MALDI-TOF	1	8.33
Enterococcus faecalis identified as Enterococcus faecium by MALDI-TOF	4	33.33
Candida tropicalis identified as Candida rugosa by MALDI-TOF	2	16.67
Candida parapsilosis identified as Candida metapsilosis by MALDI-TOF	2	16.67
Candida glabrata identified as Candida auris by MALDI-TOF	3	25.00
TOTAL	12	100.00

**Figure 3:**
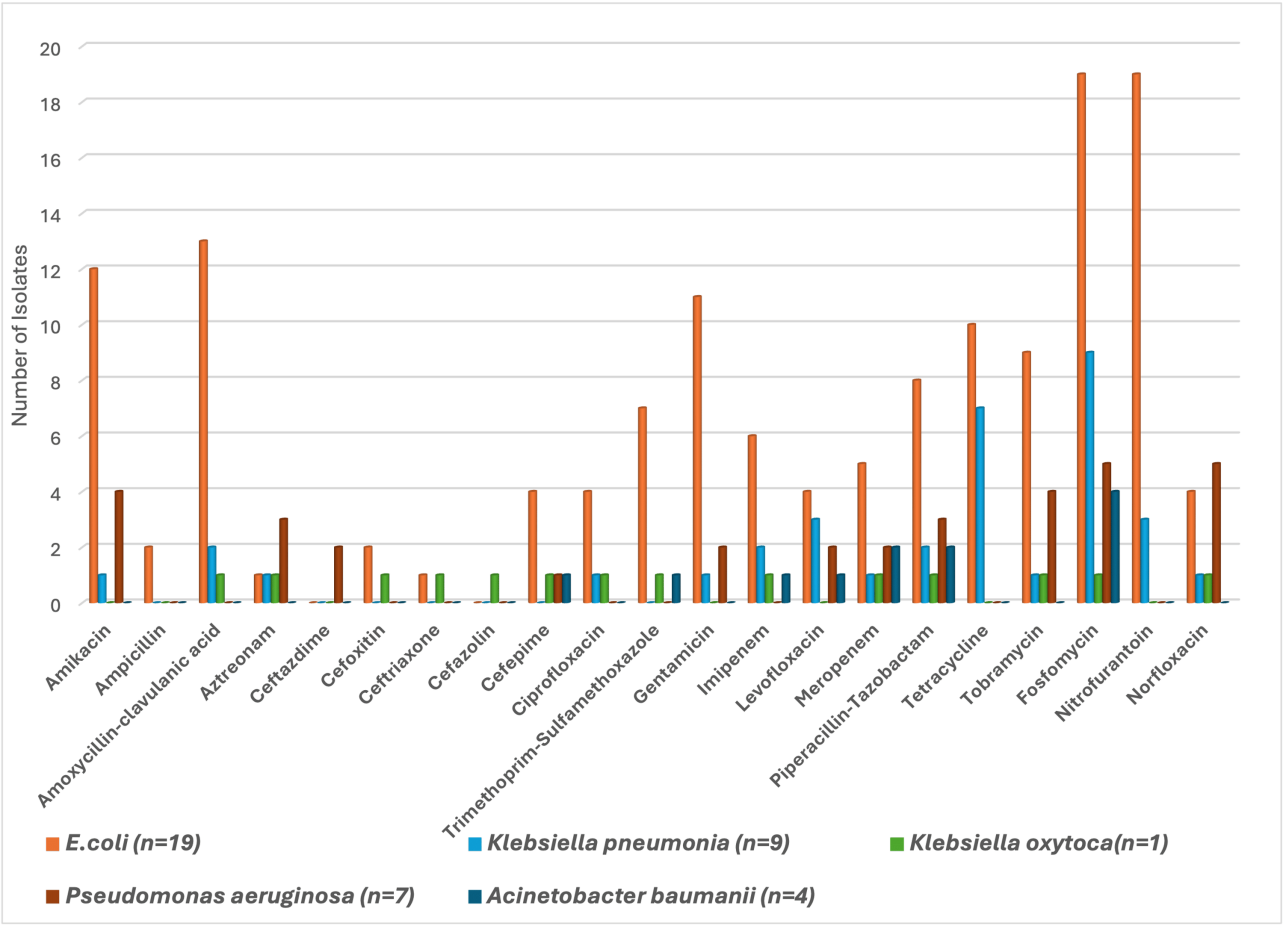
Antibiotic Sensitivity Pattern of Sensitive Gram-Negative Uropathogens in CAUTI Patients CAUTI: Catheter-Associated Urinary Tract Infection

The antimicrobial sensitivity pattern of the Gram-negative isolates is shown in Figure 3. In Escherichia coli isolates, the antibiotic nitrofurantoin as well as fosfomycin were found to be most sensitive in CAUTI patients. The antibiotic fosfomycin followed tetracycline and nitrofurantoin were found most sensitive against Klebsiella pneumoniae. Further, against Klebsiella oxytoca uropathogens, the antibiotic fosfomycin, followed by amoxicillin-clavulanic acid and ceftriaxone, was found to be sensitive. Similarly, against Pseudomonas species uropathogens, norfloxacin and fosfomycin were found to be equally effective, followed by tobramycin and amikacin in CAUTI patients. For Acinetobacter baumanii uropathogens, the antibiotic fosfomycin followed by piperacillin-tazobactam and levofloxacin were found to be sensitive.

In Enterococcus fecalis, the antibiotics linezolid, vancomycin, high-level streptomycin as well as fosfomycin were found most sensitive in CAUTI patients. Similarly, the antibiotics fosfomycin and linezolid as well as high-level streptomycin were found most sensitive against Enterococcus fecium, as shown in Table 5.

**Table 5:** Antibiotic Sensitivity Pattern of Gram-Positive Cocci Uropathogens in CAUTI Patients CAUTI: Catheter-Associated Urinary Tract Infection S – Susceptible: The microorganism is inhibited by the antibiotic at standard dosing. I – Intermediate: The microorganism may be inhibited if higher doses are used or in certain body sites. R – Resistant: The microorganism is not inhibited by the antibiotic at standard dosing, and treatment is likely ineffective.

ANTIBIOTICS	Enterococcus fecalis (n=9)						Enterococcus faecium (n=19)					
	S		I		R		S		I		R	
	N	%	N	%	N	%	N	%	N	%	N	%
Tetracycline	4	44.44	2	22.22	3	33.33	13	68.42	0	0.00	6	31.58
Levofloxacin	0	0.00	0	0.00	9	100.00	0	0.00	0	0.00	19	100.00
Ampicillin	1	11.11	0	0.00	8	88.89	0	0.00	0	0.00	19	100.00
Erythromycin	0	0.00	0	0.00	9	100.00	4	21.05	0	0.00	15	78.95
High level Gentamicin	2	22.22	0	0.00	7	77.78	5	26.32	0	0.00	14	73.68
High level Streptomycin	4	44.44	0	0.00	5	55.56	11	57.89	0	0.00	8	42.11
Ciprofloxacin	0	0.00	0	0.00	9	100.00	0	0.00	0	0.00	19	100.00
Vancomycin	9	100.00	0	0.00	0	0.00	10	52.63	0	0.00	9	47.37
Teicoplanin	8	88.89	0	0.00	1	11.11	12	63.16	0	0.00	7	36.84
Linezolid	9	100.00	0	0.00	0	0.00	19	100.00	0	0.00	0	0.00
Fosfomycin	9	100.00	0	0.00	0	0.00	19	100.00	0	0.00	0	0.00
Nitrofurantoin	3	33.33	2	22.22	4	44.44	12	63.16	0	0.00	7	36.84
Norfloxacin	1	11.11	0	0.00	8	88.89	3	15.79	0	0.00	16	84.21

## Discussion

More than five million patients in emergency critical care hospitals and long-term care homes had a urinary catheter placed [8]. They are therefore more susceptible to CAUTI and its aftereffects. Per urethral catheterization is the most significant risk factor for UTIs worldwide [9].

In this study, among the catheterized patients in the ICU, we processed 780 urine samples in which significant growth was seen in 156 (20%) samples. Similar observations were seen in a study by Vyawahare et al. where out of the 345 catheterized urine samples processed, significant growth was seen in 119 (34.4%) samples [8]. The maximum number of patients (40.4%) in our study were from the age group 18-40 years. However, previous studies have shown that patients older than 40 years are at greater risk. In a study by Jain et al., they found that the maximum patients were from the age group 40-60 (37.1%) [10]. Likewise, in a study by Vyawahare et al., the maximum number of patients (41%) were of the age group 40-60 years, while 103 (30%) patients were of the age group >60 years [8]. It can be seen from previous studies that the extremes of age are at maximum risk; this might be due to lowered defense mechanisms. The discrepancy in our study might be due to the hospital-based nature of the study.

While analyzing the risk factors of CAUTI patients, we found that the female population was significantly associated with catheter-associated UTIs (p-value: 0.017). This may be explained by anatomical differences, as females have a shorter urethra and closer proximity of the urethral opening to the anus, which facilitates bacterial entry into the urinary tract. Many studies have been conducted that support this finding. The female population was found to be a significantly associated risk factor in the study by Vywahare et al. [8]. On the contrary, some studies have also been done that describe catheter-associated UTI to be more common in the male population. Jain et al. described a study where candiduria was found more in males (61.4%) than in females (38.6%). Also, Saleem et al. reported that male patients had more CAUTI compared to female patients. This might be because of the predominance of other risk factors in their study [10,11].

We found that the most commonly observed microorganism was Candida species (56.4%). Candida tropicalis (33.33%) was predominantly isolated, followed by Candida albicans (16.67%) and Candida auris (1.92%). Enterococcus spp. was isolated in 28 (17.94%) cases followed by Escherichia coli in 19 (12.17%), Klebsiella spp. in 10 (6.41%) cases, Pseudomonas spp. in seven (4.48%) cases, and Acinetobacter baumanii in four (2.56%) cases. Similar observations were also reported by Jain et al., where they observed that Candida tropicalis (52.9%) was the predominant organism, followed by Candida albicans (28.6%) [10]. Previous studies, such as those by Koulenti et al. and Garini et al., have identified several risk factors associated with Candida urinary tract infections, including prolonged catheterization, broad-spectrum antibiotic use, diabetes mellitus, immunosuppression, and extended ICU stay. These factors likely contribute to the increased risk of infection observed in our study population [12,13].

In our study, no discrepancy was seen amongst the isolates by conventional method versus MALDI-TOF MS at the genus level. Discrepancy was found in 12 (7.7%) isolates (seven bacterial isolates and five Candida isolates) at the species level. A study by Risch et al. found a similar discrepancy of the species and/or genus in 27 samples (13.2%). The discrepancy appeared with 16 Gram-negative and 11 Gram-positive isolates [14].

Our study found that most Gram-negative isolates were resistant to commonly used antibiotics. Escherichia coli showed sensitivity to amikacin, amoxicillin-clavulanic acid, gentamicin, nitrofurantoin, and fosfomycin. Enterococcus spp. were resistant to levofloxacin, ciprofloxacin, ampicillin, erythromycin, and high-level gentamicin, but sensitive to linezolid and fosfomycin. These findings align with previous studies, such as those by Taiwo et al., Verma et al., and Sangamithra et al., which reported high resistance to antibiotics like cephalosporins and fluoroquinolones [15-17]. The trend of increased resistance, particularly to fluoroquinolones, may be due to poorly directed antibiotic prophylaxis and empiric management of CAUTI.

Limitations

This study has certain limitations. First, it was conducted at a single tertiary care center, which may limit the generalizability of the findings to other healthcare settings. Second, molecular methods such as PCR-based techniques were not included for pathogen identification, which could have provided a more comprehensive assessment of microbial diversity.

## Conclusions

Our study of 780 catheterized urine samples from ICU patients found significant bacterial growth in 156 samples (20%). Most CAUTI cases occurred in patients aged 18-40, with a higher prevalence in females, older adults, and those with diabetes mellitus.

Discrepancies in pathogen identification between conventional methods and MALDI-TOF MS were minimal but notable at the species level. These findings highlight the critical need for targeted antibiotic prophylaxis and management strategies to address the rising antibiotic resistance in CAUTI cases.
